# Measuring stress in podiatric students in Spain: psychometric validation and adaptation of the KEZKAK questionnaire

**DOI:** 10.7717/peerj.10439

**Published:** 2020-12-09

**Authors:** Ana Belen Ortega-Avila, Pablo Cervera-Garvi, Ana Maria Jimenez-Cebrian, Esther Chicharro-Luna, Irene Garcia-Paya, Gabriel Gijon-Nogueron

**Affiliations:** 1 Department of Nursing and Podiatry, University of Malaga, Malaga, Spain; 2 Department of Behavioural Sciences and Health, Faculty of Medicine, University Miguel Hernandez, San Juan de Alicante, Spain; 3 Biomedical Research Institute (IBIMA), Malaga, Spain

**Keywords:** KEZKAK, Podiatry, Reliability, Validity

## Abstract

**Objective:**

The study aim was to develop a linguistic-cultural adaptation of the KEZKAK questionnaire to be completed during the practicum of podiatric medical students in Spain, to validate the questionnaire and to evaluate its psychometric properties.

**Methods:**

The cross-sectional study was carried out in two stages: 1. Cross-cultural adaptation; 2. Clinimetric validation based on assessments of interobserver reliability, test-retest reliability and internal consistency. The participants were podiatric medical students at the universities of Malaga and Miguel Hernandez, Alicante (Spain) and were recruited during the period February–October 2019. The following inclusion criteria were applied: aged at least 18 years, studying the third or fourth year of a university degree in Podiatry. All gave signed informed consent and completed the State-Trait Anxiety Inventory and the Podiatry version of the KEZKAK questionnaire. No sampling was performed and thus the entire eligible population was included in the study.

**Results:**

The analysis was based on 205 participants (33.5% male, 66.5% female), with a mean age of 23.05 (SD 5.37) years. Internal consistency was excellent, with a Cronbach’s alpha of 0.95. This version of the questionnaire had five factorial structures (61.18%). No floor/ceiling effect was observed in any item. The KEZKAK presented high test-retest reliability after 21 days, with an overall ICC of 0.95 (95% CI [0.93–0.98]).

**Conclusion:**

For university students of podiatry in Spain, the KEZKAK Podiatry version questionnaire is a valid, reliable instrument for measuring stressors during the practicum.

## Introduction

Stress is a non-specific response of the organism to various stimuli. It is an adaptive, emergency process, and essential to survival (Román Collazo & Carlos Alberto Hernández Rodríguez, 2011). It is the generating agent of emotions and not an emotion in itself. It consists of a relationship between the individual and the environment, providing a perception of the extent to which environmental demands constitute a danger to well-being, and of whether the personal resources available are sufficient to meet these demands(Castillo Pimienta, Chacón de la Cruz & Díaz-Véliz, 2016).

In general, stress is positive at low levels, which predispose us to act against the demands presented by the environment. However, when it is moderate or extreme, stress demands greater attention, because the reactions it provokes in our ways of thinking and feeling may lead us to respond inappropriately (Alsulami et al., 2018; Marquina Luján & Pineda Palomino, 2019).

The clinical practicum is an element of vital importance in the curriculum of Health Sciences students, giving them the opportunity to perform techniques, acquire knowledge, develop skills and learn attitudes, within a clinical health environment that was previously unfamiliar and which is sometimes not as welcoming as they expect. Preparation and training prior to the student’s departure from academia to the real world is one of the great challenges of teaching, but not one to be addressed solely by the graduate student; undergraduates, too, must complete their theoretical-practical training in real environments. The presence of stress in university students is a reality. In healthcare programmes in which internships are carried out with patients in clinical situations and real environments, the stress inherent to the situation is aggravated by the psychological tension faced (Losa-Iglesias et al., 2019).

Studies have shown that, in general, health sciences students are exposed to higher levels of stress than are other students and the population in general. This stress is associated with the requirement to carry out clinical practices with real patients and in authentic clinical settings. However, most previous research in this field has focused on students of medicine and nursing (Castillo Pimienta, Chacón de la Cruz & Díaz-Véliz, 2016; Duarte et al., 2017; Losa-Iglesias et al., 2019). According to Rodrigues et al. (2016), the stress factors reported by nursing students concern feelings of insecurity, powerlessness, fear of the unknown and academic overload (Rotenstein et al., 2016). Similarly, podiatry students may experience stress in carrying out their clinical practices, where they are exposed to continuous stressors, such as fear of not doing well, doubting how to act, fear of cutting themselves or the patient with the scalpel, etc. In view of the real, stress-generating clinical scenarios faced, students must learn self-control. To help them do so, our aim is to calibrate the emotions and stress provoked in these situations.

In this line of stress measurement, a recent study evaluated the reliability and repeatability of findings obtained by the Stress Assessment in Nursing Students (ASNS) scale, modified for podiatry students, concluding that this instrument presented good psychometric properties (Palomo-López et al., 2020).

In this respect, too, the KEZKAK questionnaire was developed by Zuripia to identify stressors experienced by nursing students in their practicum (Zupiria Gorostidi et al., 2003). This questionnaire is a validated measurement instrument, for use in Spanish and Basque, that addresses the main stressful situations encountered by nursing students in their practicum, by means of direct questions in this respect.

In another investigation, the KEZKAK and other questionnaires were considered as the basis for an integrative evaluation of general self-efficacy, perceived competence, resilience and stress among nursing students, thus facilitating training in professional competencies, in line with the European guidelines of higher education. The authors suggested it would be interesting to follow up this research with comparative studies, considering different clinical contexts (Orkaizagirre-Gómara et al., 2020).

Accordingly, the aims of the present study are to develop a linguistic-cultural adaptation of KEZKAK for Spanish university students of podiatry, to be employed for measuring stressors during their practicum, to validate this adaptation and to evaluate its psychometric properties.

## Materials & Methods

### Ethical approval

This study was approved by the Medical Research Ethics Committee of the University of Malaga (CEUMA 88-2019-H) and of Miguel Hernandez University, San Juan de Alicante (DCC. ECL.01.19 PROV). The study was conducted in accordance with the Declaration of Helsinki and with all applicable ethical standards for human experimentation. Written informed consent from all participants (students) was obtained.

### Design

This observational, cross-sectional study was conducted to validate the adaptation of the KEZKAK questionnaire for Spanish university students of podiatry, for measuring stressors during their practicum.

### Setting and participants

All participants were university students of podiatry, at the University of Malaga (Spain) or the Miguel Hernandez University, Alicante (Spain), and were recruited from February to October 2019. A convenience sample was obtained of 223 students who met the criteria for inclusion: aged at least 18 years and currently studying the third or fourth year of a Bachelor’s Degree in Podiatry. International exchange students were excluded because they were not native speakers of Spanish. First and second-year students were excluded because they were considered to have insufficient theoretical clinical knowledge. No sampling was performed and thus the entire eligible population was included in the study.

### Procedure

Before undertaking the psychometric validation of this questionnaire for podiatry students, permission was obtained from the author (X. Zuripia Gorostedi).The linguistic-cultural adaptation of the original KEZKAK into the Spanish-language version for university students of Podiatry was performed by two Spanish podiatrists with nursing qualifications and university professor with PhDs, working independently (Beaton et al., 2000). The original KEZKAK questionnaire was designed to be a self-administered instrument focused on the main stressful situations encountered by nursing students in their clinical practices and available in two languages, Spanish and Basque.

In the initial questionnaire, based on 62 ideas, 55 statements were presented, based on items initially suspected of generating stress in nursing practice students. These statements were subsequently analysed and streamlined to combine those which were similar and to eliminate items not directly relevant to the project aims, resulting in a final version with 41 items. The fundamental aim of the questionnaire is to detect, according to the nursing students consulted, the most stressful aspects of their clinical practice. Among the questionnaire items, those concerning the nurses’ relationships with patients, the contact with human suffering and the tasks involved in patient care were the most representative of nurses’ concerns, rather than those concerning nurses’ relations with co-workers or the organisation of responsibilities. The students were instructed to choose one of four response options: No stress = 0, Some stress = 1, Quite a lot of stress = 2, A great deal of stress = 3. The questionnaire addresses a single main construct, but includes nine subscales referring to different types of stress. Factor 1 involves items related to fear of harm (to oneself or the patient) or of being unable to help the patient. Factor 2 refers to situations of contact with suffering; Factor 3, to the relationship with tutors and peers; Factor 4, to feelings of helplessness and uncertainty; Factor 5, to the lack of control over relationships with patients; Factor 6, to emotional involvement, both with the patient and with professional responsibilities; Factor 7, to ill-treatment or lack of consideration by the patient, and the resulting malaise; Factor 8, to behaviour suggesting the patient is seeking an intimate relationship with the student; finally, factor 9 refers to situations of work overload.

The KEZKAK questionnaire presents high internal consistency (Cronbach’s *α* = 0.95), considerable reliability (0.72 at two months and 0.68 at six months), and acceptable concurrent validity (0.39 with anxiety- feature). The factor analysis yields nine factors that have a high internal consistency and explain 64.4% of the variance.

The original questionnaire items were modified to make them appropriate for podiatry students, such as item 15 “Injuring myself with an infected needle or with a scalpel”, item 16 “Mistaking medication or treatment”, item 28 “The relationship with the podiatry student” and item 38 “Not finding the teacher/podiatrist when the situation requires it”. Moreover, the following items were eliminated: item 18 “Seeing a patient die”, item 25 “The relationship with the patient”, item 27 “Having to be with a patient’s family when the patient is dying”, item 31 “Having to be with a patient from whom bad news has been withheld” and item 39 “Having to be with a terminally ill patient”. The final adapted version was obtained after a consensus meeting between the Project leader and two independent Spanish podiatrists, all of whom are university professors with PhDs, who checked the validity of the adaptation and ensured there were no discrepancies. The comprehensive, linguistic-cultural equivalence to the podiatry terms employed was verified, after which a final version for field testing was drafted. The final version for podiatry students contained 36 items, which were pre-tested by 15 podiatry students, who confirmed the understandability of the items and of the procedure for completing the questionnaire.

### Data collection

At each of the universities involved, the students, in face-to-face sessions, provided demographic data (age, sex, employment status and marital status) and completed the State-Trait Anxiety Inventory (STAI) questionnaire and the Podiatry version of the KEZKAK questionnaire.

The STAI questionnaire is a generic instrument that is used to measure anxiety, one of the psychological disorders that is most prevalent among the general population, including university students (Spielberg, Gorsush & Lushene, 1970). STAI consists of two scales: trait anxiety (the stable tendency to perceive stimuli as threatening) and state anxiety (the presence of anxiety-provoking stimuli in the environment during the period shortly before the evaluation). This questionnaire was included with the KEZKAK to test the convergent validity. The STAI is scored on a scale ranging from 0 to 60 points, in which the higher the score, the greater the anxiety. This questionnaire has been translated into Spanish and is validated for use in this language (Buela-Casal, Guillén-Riquelme & Seisdedos Cubero, 2011). The podiatry version of the KEZKAK questionnaire contains 36 items with four response options (No stress = 0, Some stress = 1, Quite a lot of stress = 2, A great deal of stress = 3).

### Statistical analysis

All statistical analyses were performed with IBM SPSS Statistics for Windows, Version 25.0. (Armonk, NY: IBM Corp.). Data cleansing was performed by item analysis, applying a corrected item-total correlation of <0.5, unless the item referred expressly to the context of podiatry. Descriptive statistics of the variables were obtained, and the normality of their distribution was confirmed by the Kolmogorov–Smirnov test. Spearman’s test correlations were determined, according to normality. All calculations were performed assuming a 95% confidence interval (95%CI). A test reading was performed, using the Flesch Kincaid Grade Level and the Flesch Reading Ease tests, to assess the readability of the questionnaire (Centers for Disease Control and Prevention, 2009). Content validity was assessed by the distribution of the scores and by the presence/absence of a floor-ceiling effect, according to the endorsement frequency, with a maximum accepted value of 15%.

For the clinimetric validation, interobserver reliability and test-retest reliability were evaluated using Pearson correlation coefficients and intraclass correlation coefficients (ICC). These tests were administered twice by the same observer, at an interval of 21days, and the results obtained were classed as >0.7 “excellent”, 0.60–0.74 “good”, 0.40–0.59 “fair” and <0.40 “poor”(Shrout & Fleiss, 1979). Internal consistency was assessed by Cronbach’s alpha, for which values of 0.7, 0.8 and 0.9 were considered to represent fair, good and excellent degrees of internal consistency, respectively. Construct validity was assessed by confirmatory factorial analysis with Varimax rotation. Convergent validity was assessed by testing the relationship between the Spanish-language version of KEZKAK for podiatry students and the STAI (Terwee et al., 2007).

## Results

A total of 223 students were initially recruited to this study. After application of the inclusion and exclusion criteria, 205 third and fourth-year students from the universities concerned finally completed the questionnaires and were included in the analysis. Of these students, 33.5% were male and 66.5% female, with an average age of 23.05 (SD 5.37) years. Only 22.3% were economically active, and their work activity was not related to health care (Table 1).

**Table 1:** Sociodemographic characteristics.

		*n* (206)	Frequency (%)
Sex	Male	69	33.5
	Female	137	66.5
In employment	No	160	77.7
	Yes	46	22.3
Marital status	Single	197	95.6
	Married	5	2.4
	Divorced	4	1.9
		Mean	SD
Age		23.05	5,376

### Linguistic-cultural adaptation

A linguistic-cultural adaptation of the KEZKAK questionnaire was developed to enable its Spanish-language use with university students of Podiatry. The participants all understood the items in the final questionnaire without requiring any kind of interpretation or assistance. The data obtained were digitised and then cleansed by application of the following threshold for item elimination: total/corrected item correlation <0.4. As a result of this exercise, items K1, K2 and K32, corresponding to “Not feeling part of the work team”, “Doing my job incorrectly and harming the patient” and “Receiving contradictory orders”, respectively, were eliminated from the questionnaire. The Flesch Reading Ease and Flesch Kincaid Grade Level tests were then applied, producing results of 57.4 and 7.9, respectively.

### Reliability

The adapted questionnaire obtained excellent internal consistency, with a Cronbach’s alpha of 0.953. The inter-item correlation matrices are shown in Table 2.

**Table 2:** The inter-item correlation matrices.

	K3	K4	K5	K6	K7	K8	K9	K10	K11	K12	K13	K14	K15	K16	K17	K18	K19	K20	K21	K2	K23	K24	K25	K26	K27	K28	K29	K30	K31	K33	K34	K35	K36
K3																															
K4	0.72																																
K5	0.68	0.69																															
K6	0.67	0.62	0.65																														
K7	0.60	0.60	0.64	0.68																													
K8	0.38	0.39	0.41	0.38	0.37																												
K9	0.46	0.43	0.40	0.45	0.48	0.52																											
K10	0.41	0.46	0.44	0.35	0.44	0.56	0.61																										
K11	0.28	0.32	0.29	0.31	0.33	0.43	0.35	0.46																									
K12	0.30	0.40	0.28	0.27	0.35	0.43	0.34	0.53	0.47																								
K13	0.39	0.44	0.31	0.35	0.40	0.34	0.40	0.34	0.45	0.38																							
K14	0.40	0.45	0.42	0.38	0.41	0.38	0.49	0.43	0.46	0.40	0.45																						
K15	0.32	0.39	0.32	0.32	0.39	0.42	0.41	0.32	0.48	0.43	0.65	0.55																					
K16	0.51	0.46	0.42	0.40	0.45	0.37	0.41	0.29	0.29	0.32	0.47	0.58	0.57																				
K17	0.44	0.36	0.46	0.44	0.53	0.33	0.32	0.30	0.37	0.28	0.37	0.40	0.46	0.60																			
K18	0.32	0.33	0.21	0.24	0.35	0.31	0.28	0.35	0.44	0.59	0.39	0.41	0.36	0.30	0.29																		
K19	0.48	0.40	0.48	0.48	0.50	0.36	0.32	0.27	0.39	0.31	0.42	0.50	0.48	0.51	0.57	0.40																	
K20	0.26	0.32	0.22	0.21	0.30	0.39	0.25	0.39	0.30	0.41	0.27	0.32	0.32	0.19	0.21	0.35	0.31																
K21	0.34	0.30	0.33	0.33	0.31	0.40	0.33	0.42	0.33	0.42	0.33	0.38	0.33	0.29	0.31	0.35	0.30	0.45															
K22	0.39	0.36	0.37	0.28	0.36	0.45	0.38	0.41	0.46	0.46	0.46	0.54	0.50	0.45	0.37	0.44	0.54	0.35	0.48														
K23	0.30	0.36	0.31	0.26	0.25	0.35	0.30	0.40	0.65	0.43	0.46	0.44	0.48	0.35	0.34	0.44	0.42	0.33	0.34	0.55													
K24	0.42	0.45	0.36	0.43	0.39	0.38	0.46	0.40	0.49	0.36	0.52	0.57	0.58	0.60	0.51	0.43	0.53	0.28	0.36	0.52	0.55												
K25	0.26	0.36	0.24	0.16	0.28	0.39	0.31	0.49	0.36	0.56	0.36	0.36	0.68	0.30	0.24	0.48	0.23	0.43	0.52	0.43	0.34	0.39											
K26	0.42	0.34	0.38	0.45	0.41	0.38	0.50	0.43	0.32	0.39	0.30	0.340	0.41	0.33	0.37	0.28	0.44	0.35	0.48	0.47	0.29	0.46	0.38										
K27	0.26	0.26	0.18	0.23	0.29	0.24	0.22	0.33	0.42	0.31	0.33	0.39	0.37	0.25	0.27	0.27	0.34	0.32	0.42	0.38	0.41	0.42	0.42	0.41									
K28	0.33	0.31	0.30	0.45	0.37	0.37	0.46	0.45	0.30	0.31	0.24	0.39	0.33	0.27	0.34	0.23	0.40	0.33	0.49	0.46	0.26	0.43	0.38	0.61	0.44								
K29	0.40	0.43	0.44	0.43	0.47	0.31	0.29	0.35	0.38	0.37	0.30	0.41	0.26	0.37	0.46	0.34	0.43	0.36	0.36	0.40	0.31	0.45	0.37	0.47	0.36	0.51							
K30	0.20	0.28	0.24	0.26	0.23	0.28	0.38	0.34	0.44	0.34	0.33	0.4	0.43	0.37	0.29	0.23	0.36	0.32	0.41	0.39	0.53	0.59	0.33	0.47	0.54	0.45	0.47						
K31	0.23	0.25	0.13	0.16	0.29	0.20	0.26	0.32	0.39	0.41	0.39	0.33	0.39	0.27	0.28	0.43	0.34	0.4	0.44	0.43	0.39	0.44	0.42	0.37	0.45	0.33	0.35	0.47					
K33	0.32	0.30	0.29	0.25	0.32	0.37	0.39	0.37	0.54	0.46	0.40	0.47	0.52	0.43	0.34	0.41	0.44	0.28	0.36	0.56	0.55	0.52	0.36	0.42	0.37	0.31	0.35	0.53	0.41				
K34	0.39	0.40	0.34	0.36	0.40	0.28	0.32	0.37	0.39	0.37	0.38	0.41	0.40	0.33	0.35	0.35	0.46	0.36	0.35	0.43	0.37	0.40	0.38	0.48	0.43	0.48	0.43	0.38	0.46	0.46			
K35	0.31	0.38	0.29	0.35	0.26	0.27	0.40	0.35	0.41	0.31	0.43	0.51	0.45	0.42	0.28	0.37	0.35	0.24	0.39	0.45	0.49	0.66	0.26	0.39	0.27	0.32	0.32	0.50	0.40	0.58	0.41		
K36	0.27	0.23	0.21	0.23	0.26	0.20	0.12	0.22	0.34	0.33	0.35	0.37	0.31	0.26	0.28	0.30	0.40	0.34	0.44	0.43	0.34	0.33	0.32	0.37	0.40	0.29	0.31	0.25	0.43	0.32	0.51	0.23	

### Test-retest validity

No ceiling/floor effect was observed for any item. The KEZKAK questionnaire presented high test-retest reliability after 21 days, with an overall ICC of 0.95 (95% CI [0.93–0.98]).

### Construct validity

**Figure 1:**
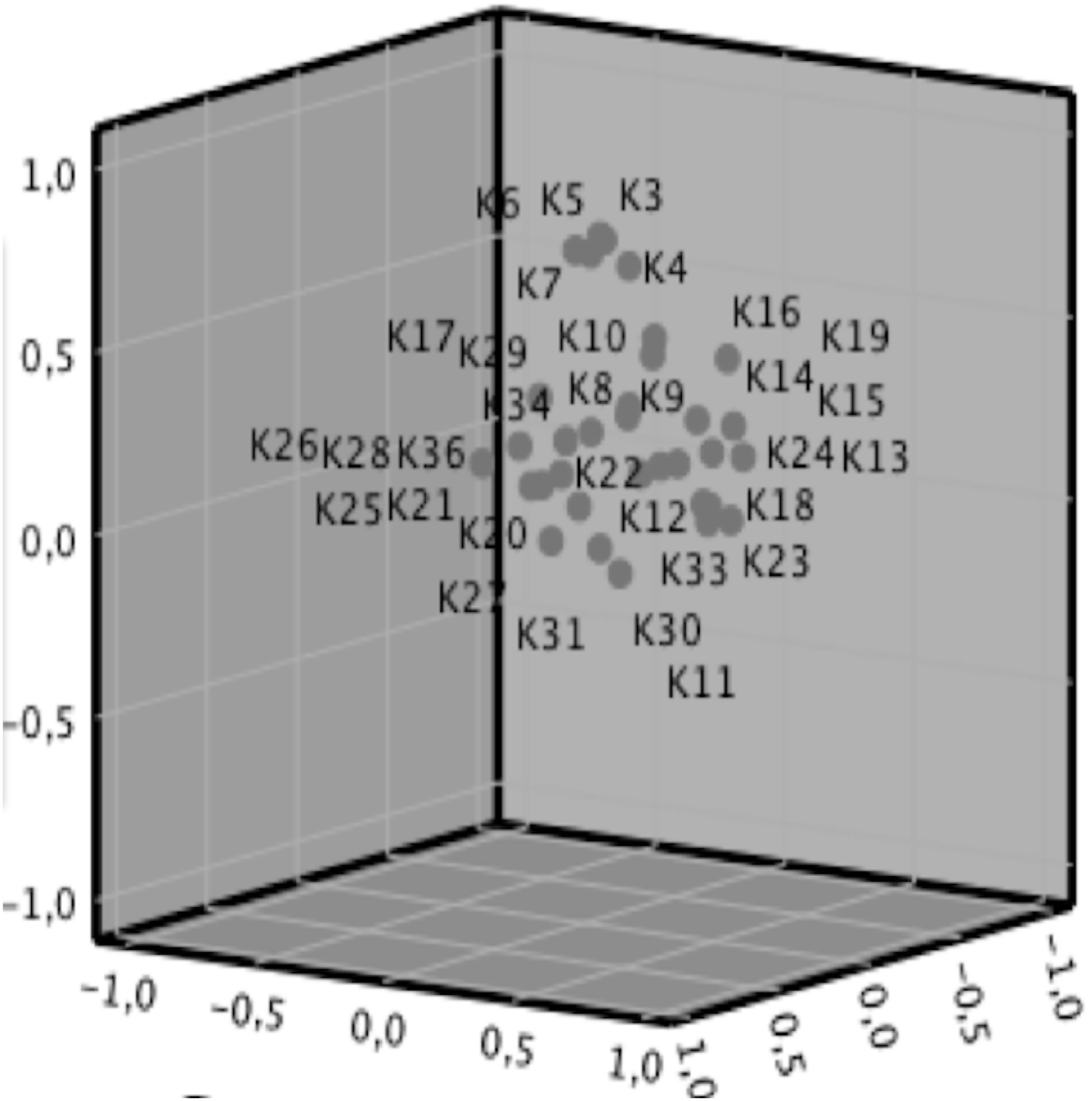
Component plot in rotated space. The left view is Component 1, the front view is Component 2, and the right view is Component 3.

Factor analysis was conducted by application of the Kaiser–Meyer-Olkin and Bartlett tests. The adapted questionnaire was found to have five factorial structures (Fig. 1). According to total variance analysis, the Podiatry version of KEZKAK accounted for 61.185% of total variance.

### Convergent validity

The correlation with the STAI was weak, but statistically significant (*p* < 0.05) (Table 3).

**Table 3:** Convergent validity.

**Convergent Validity**			
Rho Spearman	State Anxiety		0.204
		*P* value	0.003^*^
		Number	205
	Trait Anxiety		0.034
		*P* value	0.021
		Number	205

**Notes.**

* P value ≤ 0.05

## Discussion

The aim of this study was to develop a cultural-linguistic adaptation of the KEZKAK questionnaire to be completed by Spanish university students of podiatry, as a means of measuring stressors during their practicum. We then evaluated this questionnaire and its psychometric properties. The results obtained show that the instrument tested is valid and reliable for this target population.

The study population were all volunteers and homogeneous in terms of age, although by sex there was a predominance of female students (66.5%), presumably due to their greater interest in university studies related to the health sciences (García-Díaz et al., 2020). This gender imbalance is present in undergraduate nursing programmes worldwide, and has been reported in many national and international studies (Boostel et al., 2018). The linguistic and cultural adaptation was performed with little difficulty following Beaton’s recommendations (Beaton et al., 2000).

Five of the initial items were unrelated to the context of podiatry students (such as item 18, “Seeing a patient die”) and so were excluded from the questionnaire. After filtering the items, the instrument finally obtained consisted of a self-administered questionnaire with 33 items, 5 factors, internal consistency, test re-test reliability, item-total and inter-item correlation and convergent validity to the STAI.

The Podiatry questionnaire presented five factors, in contrast to the nine of the original version, However, factorial analysis of the former showed that these five factors accounted for 61.185% of the total variance, a very similar level to that of the original, which had nine factors (64.4% of the total variance). These findings support the assumption of construct validity and show that the adapted questionnaire can safely be used to assess the presence and impact of stressors during the practicum of podiatry students. The first factor (17.22%) was composed of items related to feelings of powerlessness in the relationship with the patient. The second (32.99%) concerned the student’s perceived lack of professional skills. The third (44.51%) was related to emotional involvement and uncertainty, the fourth (55.96%) to relationships with tutors and peers, and the fifth (61.18%), to the student’s ability to relate to patients when they were given bad news or were suffering.

The difference in factorial structure between the adapted questionnaire and the original version may have arisen from inherent differences in the two branches of university studies (Nursing vs. Podiatry). Although both correspond to the field of health sciences, they differ regarding the clinical practices required and the relationship and degree of involvement with the patient during daily practice (Castello et al., 2019; Sánchez-García et al., 2019; Christensen et al., 2020). In this respect, it should be noted that the Portuguese version of KEZKAK, for Nursing students, only contains 31 items, divided into eight stressors (Barroso et al., 2008) of which the factors “Powerlessness and uncertainty”, “Not controlling the relationship with the patient” and “The patient seeks an intimate relationship” were combined into a single factor in the podiatric version of the questionnaire. The factors “Contact with suffering” and “Malaise from relationship with the patient” were also combined.

Strong internal consistency was observed in our questionnaire, as reflected in the Cronbach’s alpha score of 0.95, which is in line with the original and Portuguese-language versions (*α* = 0.95 and *α* = 0.935, respectively). No floor or ceiling effects were observed. The Podiatry version of KEZKAK presented convergent validity, with weak correlation for the STAI, which is in line with the original version and indicates that this questionnaire is capable of identifying stressors encountered during the practicum of podiatry students. In other words, it does not measure the degree of stress experienced by the student, but identifies potentially stressful situations (Alzayyat & Al-Gamal, 2014). The previous experiences of each student could affect the perception of stressors as a threat, either generating negative emotions such as fear, anxiety or anger, or causing stress to be viewed as a challenge to be coped with and/or overcome. From an educational standpoint, this instrument can help planners and students cope with stress-inducing situations and identify possible solutions.

In the field of podiatry, the only measurement instrument comparable with ours is the Spanish-language version of the ASNS instrument for podiatric students. However, this is used to mediate stress factors, not the situations that may provoke stress, such as clinical practices with real patients during the practicum. The ASNS instrument presents good reliability (*α* = 0.883), but falls short of the excellent internal consistency we obtained (*α* = 0.95). The ASNS instrument consists of 30 items and six domains, in contrast to the Spanish-language version of KEZKAK, adapted for podiatric students, which consists of five factors and explains 61.185% of the total variance. No factor analysis was performed in the ASNS adaptation, and so the number of domains might have been modified. Neither was the respondent population fully identified (i.e., whether the students were currently in the first, second, third, …year of their course), and so it is unclear the extent to which these students had previously had contact with the clinical practices of podiatry.

The main limitation of the present study is the male–female imbalance of the study population, which made it necessary to normalise the sample. In addition, in the cross-cultural validation, the issues related to validity should be assessed by the expert panel. Moreover, further items related to clinical practice could have been added to the questionnaire. On the other hand, in the validation that was performed, after the initial digitisation it was necessary to perform data cleansing, which led to some items being excluded, in part due to the real-life practice of podiatry in Spain which tends to be not in specialist or multidisciplinary teams, but often involves working alone.

In future research, it would be useful to observe the behaviour of stressors over time, in a longitudinal study, and also to conduct a quantitative evaluation of our questionnaire. Nevertheless, it is unarguable that clinical simulation for students is a necessary part of their preparation, and that this allows students to experience a close-to-real-life situation, highlighting the importance of scientific knowledge and skill, and their responsibility to provide the podiatry care that patients need. In addition, it would be useful to expand the focus to consider other podiatry-teaching institutions in Spain and/or the rest of the world, and to consider other branches of university study within the health sciences, where practicums with patients take place, to determine whether these present a similar pattern of reaction to stress. Such a focus would provide a broader perspective, help identify the main stressors experienced and enable us to design and apply strategies to decrease their intensity, thus enhancing students’ satisfaction with their education and promoting high-quality, safe healthcare. Finally, students who have completed their studies in other branches of health science should be evaluated to determine whether the impact of stressors decreases with greater experience.

The main strength of the present study is that it introduces the first self-administered questionnaire that can be used to measure the stressful situations encountered by university students of Podiatry in their practicum. Moreover, it contributes to our understanding of podiatry by showing that the main stressors that affect students are related to a perceived lack of competence and to difficulties in relationships. The findings presented, therefore, represent documentary support for teachers and course designers in the field of podiatry with which to create strategies aimed at reducing the factors shown to aggravate stress among these students.

## Conclusions

The podiatry version of the KEZKAK is a self-administered questionnaire that is valid and reliable for measuring stressors during students’ practicum. However, further analysis may be needed, examining the use of this instrument with a larger population and incorporating a longitudinal follow-up. The use of this questionnaire is expected to improve students’ capacity for self-evaluation and critical reflection concerning their responsibility to learn and to acquire the healthcare skills needed.

##  Supplemental Information

10.7717/peerj.10439/supp-1 Supplemental Information 1 General data and data from the questions in the KEZKAK questionnaire collected by the students and the STAI questionnaire that is used as the gold standard Rows: students; columns: data. Gender (man = 0; woman = 1) age; job (yes = 1, no = 0); K (number of questionnaire questionnaire); SAE (number of STAI questionnaire); FAC (Confirmatory Analysis Factor)

10.7717/peerj.10439/supp-2 Supplemental Information 2 Codebook to convert numbers to their respective factors

10.7717/peerj.10439/supp-3 Supplemental Information 3 Kezkak questionnaire and the English translation.

10.7717/peerj.10439/supp-4 Supplemental Information 4 Stai questionnaire and the English translation.

## Additional Information and Declarations

### Data Availability

The following information was supplied regarding data availability: The raw database is available in the Supplementary Files.
