# Supplementary material for: Measuring stress in podiatric students in Spain: psychometric validation and adaptation of the KEZKAK questionnaire
Source: PeerJ. 2020 Dec 9;8:e10439. doi: 10.7717/peerj.10439 (PMC7733331; doi:10.7717/peerj.10439)
Supplement: Supplemental Information 2 [file peerj-08-10439-s002.docx]

DATABASE CODE

GENDER: man= 0; woman=1

AGE: years

JOB: No= 0; yes= 1

K (KEZKAK question): No stress = 0, Some stress = 1, Quite a lot of stress = 2, A great deal of stress = 3

SAR (STAI question) number 1-20: 0=nothing; 1=something; 2= quite; 3=a lot

SAR (STAI question) number 21-40: 0= never; 1=sometimes; 2=often; 3=usually

FAC: CONFIRMATORY ANALYSIS FACTOR
