# Supplementary material for: Measuring stress in podiatric students in Spain: psychometric validation and adaptation of the KEZKAK questionnaire
Source: PeerJ. 2020 Dec 9;8:e10439. doi: 10.7717/peerj.10439 (PMC7733331; doi:10.7717/peerj.10439)
Supplement: Supplemental Information 3 [file peerj-08-10439-s003.docx]

**KEZKAK (Adaptación a Podología)**

A continuación encontrarás una serie de enunciados que se relacionan con la preocupación al enfrentarte a las prácticas clínicas del Grado en Podología. Te solicitamos que leas y marques con una cruz el grado en que te identificas con las afirmaciones, utilizando las siguientes opciones:

**0**-NADA **1**-ALGO **2**-BASTANTE **3**-MUCHO

Por favor no dejes preguntas sin contestar

| **CUESTIONARIO KEZKAK ADAPTADO A PODOLOGÍA** | | **NADA** | **ALGO** | **BASTANTE** | **MUCHO** |
| --- | --- | --- | --- | --- | --- |
|  | No sentirme integrado/a en el equipo de trabajo |  |  |  |  |
|  | Hacer mal mi trabajo y perjudicar al paciente |  |  |  |  |
|  | Sentir que no puedo ayudar al paciente |  |  |  |  |
|  | Hacer daño psicológico al paciente |  |  |  |  |
|  | No saber cómo responder a las expectativas de los pacientes |  |  |  |  |
|  | Hacer daño físico al paciente |  |  |  |  |
|  | No saber cómo responder al paciente |  |  |  |  |
|  | Que me afecten las emociones del paciente |  |  |  |  |
|  | Tener que dar malas noticias |  |  |  |  |
|  | Tener que hablar con el paciente de su sufrimiento |  |  |  |  |
|  | Que el paciente me trate mal |  |  |  |  |
|  | La relación con los profesionales de la salud |  |  |  |  |
|  | Contagiarme a través del paciente |  |  |  |  |
|  | Que un paciente que estaba mejorando comience a empeorar |  |  |  |  |
|  | Pincharme con una aguja infectada o bisturí |  |  |  |  |
|  | Confundirme de medicación/tratamiento |  |  |  |  |
|  | "Meter la pata" |  |  |  |  |
|  | La relación con el profesor/a responsable de prácticas de la facultad |  |  |  |  |
|  | Encontrarme en una situación sin saber qué hacer |  |  |  |  |

TRANSLATED KEZKAK (Adaptation to Podiatry)

Below you will find a series of statements that relate to concern when facing the clinical practices of the Degree in Podiatry. We ask you to read and mark with a cross the degree to which you identify with the affirmations, using the following options:
0-NOTHING 1-SOMETHING 2-ENOUGH 3-A LOT
Please don't leave questions unanswered

KEZKAK QUESTIONNAIRE ADAPTED TO PODOLOGY NOTHING SOMETHING ENOUGH A LOT
1. Not feeling integrated in the work team
2. Do my job wrong and harm the patient
3. Feeling like I can't help the patient
4. Do psychological harm to the patient
5. Not knowing how to respond to patient expectations
6. Do physical harm to the patient
7. Not knowing how to respond to the patient
8. Let the patient's emotions affect me
9. Having to give bad news
10. Having to talk to the patient about their suffering
11. That the patient treats me badly
12. The relationship with health professionals
13. Spread through the patient
14. Let a patient who was getting better start to get worse
15. Prick me with an infected needle or scalpel
16. Get confused about medication / treatment
17. "screw up"
18. The relationship with the professor responsible for the faculty practices
19. Finding myself in a situation without knowing what to do
20. Getting too involved with the patient
21. That my responsibility for patient care is important
22. Not being able to reach all patients
23. That the patient does not respect me
24. Receive a patient's complaint
25. Relationship with fellow podiatry students
26. Meeting emergency situations
27. Having to be with a patient with whom it is difficult to communicate
28. Having to perform procedures that hurt the patient
29. Not knowing how to "cut" the patient
30. Having to work with aggressive patients
31. Work overload
32. Receive conflicting orders
33. That a patient of the other sex insinuates me
34. Not finding the teacher / podiatrist when the situation requires it
35. Have the patient touch certain parts of my body
36. The differences between what we learn in class and what we see in practice
