# Supplementary material for: Measuring stress in podiatric students in Spain: psychometric validation and adaptation of the KEZKAK questionnaire
Source: PeerJ. 2020 Dec 9;8:e10439. doi: 10.7717/peerj.10439 (PMC7733331; doi:10.7717/peerj.10439)
Supplement: Supplemental Information 4 [file peerj-08-10439-s004.docx]

**STAI**

**AUTOEVALUACION A (E/R)**

**A/E PD = 30 + = A/R PD = 21 + =**

**APELLIDOS Y NOMBRES :**

**EDAD: SEXO: ( V ) ( M ) FECHA:**

**CENTRO : ESTADO CIVIL :**

**A-E**

**INSTRUCCIONES**

**A continuación encontrará unas frases que se utilizan corrientemente para describirse uno a sí mismo. Lea cada frase y señale la puntuación 0 a 3 que indique mejor cómo se SIENTE UD. AHORA MISMO, en este momento. No hay respuestas buenas ni malas.**

**No emplee demasiado tiempo en cada frase y conteste señalando la respuesta que mejor describa su situación presente**

|  | Nada | Algo | Bastante | Mucho |
| --- | --- | --- | --- | --- |
| **1. Me siento calmado.** | **0** | **1** | **# 2** | **3** |
| **2. Me siento seguro.** | **0** | **1** | **# 2** | **3** |
| **3. Estoy tenso** | **0** | **1** | **# 2** | **3** |
| **4. Estoy contrariado.** | **0** | **1** | **# 2** | **3** |
| **5. Me siento cómodo (estoy a gusto).** | **0** | **1** | **# 2** | **3** |
| **6. Me siento alterado.** | **0** | **1** | **# 2** | **3** |
| **7. Estoy preocupado ahora por posibles desgracias futuras** | **0** | **1** | **# 2** | **3** |
| **8. me siento descansado** | **0** | **1** | **# 2** | **3** |
| **9. Me siento angustiado.** | **0** | **1** | **# 2** | **3** |
| **10. Me siento confortable** | **0** | **1** | **# 2** | **3** |
| **11. tengo confianza en mi mismo.** | **0** | **1** | **# 2** | **3** |
| **12. Me siento nevioso** | **0** | **1** | **# 2** | **3** |
| **13. Estoy desasosegado.** | **0** | **1** | **# 2** | **3** |
| **14. Me siento muy "atado" (como oprimido)** | **0** | **1** | **# 2** | **3** |
| **15. Estoy relajado** | **0** | **1** | **# 2** | **3** |
| **16. Me siento satisfecho** | **0** | **1** | **# 2** | **3** |
| **17. Estoy preocupado.** | **0** | **1** | **# 2** | **3** |
| **18. Me siento aturdido y sobreexitado.** | **0** | **1** | **# 2** | **3** |
| **19. Me siento alegre.** | **0** | **1** | **# 2** | **3** |
| **20. En este momento me siento bien** | **0** | **1** | **# 2** | **3** |

**COMPRUEBESIHACONTESTADO ATODASLASFRASESCONUNASOLARESPUESTA**

**Ahora, vuelva la hoja y lea las instrucciones antes de comenzar a contestar las frases.**

**A- R**

**INSTRUCCIONES**

**A continuación encontrará unas frases que se utilizan corrientemente para describirse uno a sí mismo. Lea cada frase y señale la puntuación 0 a 3 que indique mejor cómo se SIENTE UD. EN GENERAL, en la mayoría de las ocasionesq. No hay respuestas buenas ni malas. No emplee demasiado tiempo en cada frase y conteste señalando la respuesta que mejor describa su situación presente**

**Casi A A Casi**

Nunca Veces Menudo Siempre

**21 Me siento calmado. 0 1 # 2 3**

1. **Me Canso rápidamnte 0 1 # 2 3**
2. **Siento ganas de llorar 0 1 # 2 3**
3. **Me gustaría ser feliz como otros 0 1 # 2 3**
4. **pierdo oportunidades por no decidirme pronto. 0 1 # 2 3**
5. **M siento descansado 0 1 # 2 3**
6. **Soy una persona tranquila serena y sosegada 0 1 # 2 3**
7. **Veo que las dificultades se amontonan y no puedo con ellas 0 1 # 2 3**

**29. Me preocupo demasiado por cosas sin importancia 0 1 # 2 3**

1. **Soy feliz 0 1 # 2 3**
2. **Suelo tomar las cosas demasiado seriamente. 0 1 # 2 3**
3. **Me falta confianza en mi mismo 0 1 # 2 3**
4. **Me siento seguro 0 1 # 2 3**
5. **No suelo afrontar crisis o dificultades 0 1 # 2 3**
6. **Me siento triste (melancólico). 0 1 # 2 3**
7. **Estoy satisfecho. 0 1 # 2 3**
8. **Me rondan y molestan pensamientos sin importancia 0 1 # 2 3**
9. **Me afectan tanto los desengaños que no puedo olvidarlos 0 1 # 2 3**
10. **Soy una persona estable. 0 1 # 2 3**
11. **Cuando pienso sobre asuntos y preocupaciones actuales, 0 1 # 2 3**

**me pongo tenso y agitado**

**COMPRUEBESIHACONTESTADO ATODASLASFRASESCONUNASOLARESPUESTA**

TRANSLATED STAI

SELF-ASSESSMENT A (E / R) A / E PD = 30 + =

A / R PD = 21 + =

SURNAMES AND NAMES :

AGE: SEX: (V) (M) DATE:

CENTER: CIVIL STATUS:

A-E

INSTRUCTIONS

Here are some commonly used phrases to describe yourself. Read each sentence and point to the score 0 to 3 that best indicates how YOU FEEL. RIGHT NOW, right now. There is no right or wrong answers.

Don't spend too much time on each sentence and answer by pointing to the answer that best describes your current situation

Not at all Somewhat Quite a lot

1. I feel calm. 0 1 # 2 3

2. I feel safe. 0 1 # 2 3

3. I am tense 0 1 # 2 3

4. I am disappointed. 0 1 # 2 3

5. I feel comfortable (I am comfortable). 0 1 # 2 3

6. I feel upset. 0 1 # 2 3

7. I am concerned now about possible future misfortunes 0 1 # 2 3

8. I feel rested 0 1 # 2 3

9. I feel distressed. 0 1 # 2 3

10. I feel comfortable 0 1 # 2 3

11. I have confidence in myself. 0 1 # 2 3

12. I feel snowy 0 1 # 2 3

13. I am uneasy. 0 1 # 2 3

14. I feel very "tied up" (as oppressed) 0 1 # 2 3

15. I am relaxed 0 1 # 2 3

16. I am satisfied 0 1 # 2 3

17. I am concerned. 0 1 # 2 3

18. I feel groggy and over-excited. 0 1 # 2 3

19. I feel happy. 0 1 # 2 3

20. I feel good right now 0 1 # 2 3

CHECK ANY ANSWER TO ALL PHRASES WITH NO SOLAR RESPONSE

Now turn the sheet over and read the instructions before you start answering the sentences.

A- R

INSTRUCTIONS

Here are some commonly used phrases to describe yourself. Read each sentence and point to the score 0 to 3 that best indicates how YOU FEEL. IN GENERAL, in most cases q. There is no right or wrong answers. Don't spend too much time on each sentence and answer by pointing to the answer that best describes your current situation

Almost A A Almost

Never Times Often Always

21 I feel calm. 0 1 # 2 3

22. I get tired quickly 0 1 # 2 3

23. I feel like crying 0 1 # 2 3

24. I would like to be happy like others 0 1 # 2 3

25. I lose opportunities for not deciding soon. 0 1 # 2 3

26. I feel rested 0 1 # 2 3

27. I am a calm, serene and calm person 0 1 # 2 3

28. I see that the difficulties are piling up and I cannot cope with them 0 1 # 2 3

129. I worry too much about unimportant things 0 1 # 2 3

30. I am happy 0 1 # 2 3

31. I usually take things too seriously. 0 1 # 2 3

32. I lack confidence in myself 0 1 # 2 3

33. I feel safe 0 1 # 2 3

34. I don't usually face crisis or difficulties 0 1 # 2 3

35. I feel sad (melancholic). 0 1 # 2 3

36. I am satisfied. 0 1 # 2 3

37. I am haunted and bothered by unimportant thoughts 0 1 # 2 3

38. The disappointments affect me so much that I cannot forget them 0 1 # 2 3

39. I am a stable person. 0 1 # 2 3

40. When I think about current issues and concerns, 0 1 # 2 3

I get tense and agitated

CHECK ANY ANSWER TO ALL PHRASES WITH NO SOLAR RESPONSE
